# Compressive adaptive computational ghost imaging

**DOI:** 10.1038/srep01545

**Published:** 2013-03-26

**Authors:** Marc Aβmann, Manfred Bayer

**Affiliations:** 1 Experimentelle Physik 2, Technische Universität Dortmund, 44221 Dortmund, Germany; 2 Current address: JILA, University of Colorado, Boulder, CO 80309, USA.

## Abstract

Compressive sensing is considered a huge breakthrough in signal acquisition. It allows recording an image consisting of *N*^2^ pixels using much fewer than *N*^2^ measurements if it can be transformed to a basis where most pixels take on negligibly small values. Standard compressive sensing techniques suffer from the computational overhead needed to reconstruct an image with typical computation times between hours and days and are thus not optimal for applications in physics and spectroscopy. We demonstrate an adaptive compressive sampling technique that performs measurements directly in a sparse basis. It needs much fewer than *N*^2^ measurements without any computational overhead, so the result is available instantly.

Computational ghost imaging (CGI) is a novel imaging technique that has received significant attention during the last few years^1^. It is a consequent further development of conventional ghost imaging^2^,^3^ which allows to record spatially resolved images using a detector without spatial resolution. In conventional ghost imaging the image is recorded using two spatially correlated light beams, one object and one reference beam. The object beam illuminates the object to be imaged and is then collected using a bucket detector. The reference beam never interacts with the object and is recorded using a pixelated device offering spatial resolution. As both beams are spatially correlated the coincidence count signal allows one to retrieve a ghost image of the object. Ghost imaging using both entangled photons^2^ or classical light^4^,^5^,^6^ as the spatially correlated twin beam source has been demonstrated. A seminal paper by Shapiro^7^ clarified that the sole purpose of the reference beam lies in determining the illumination pattern at the object position at each instant, while the object beam gives data about the transmission of this pattern through the object. Therefore, if one can create a deterministic illumination pattern at the object position, the reference beam becomes obsolete and CGI using just a single beam and a single pixel detector^8^ becomes possible. This approach has been realized using deterministic speckle patterns created using a spatial light modulator (SLM)^9^. It has also been demonstrated that this technique also offers the possibility to perform compressive sensing^10^,^11^, that is it is possible to reconstruct an image consisting of *N*^2^ pixels using much less than *N*^2^ measurements by utilizing the fact that natural images are typically sparse^12^: When transformed to an appropriate basis, they contain many coefficients that are zero or close to it. In practice, the transmission measured for each speckle pattern constitutes a projection of the object image and compressive sensing is performed by utilizing an algorithm which checks all the possible images which are consistent with the projections performed and finds the image which is the sparsest one. Usually the *L*1-norm serves as a measure of sparsity and the image which minimizes it, is the optimal reconstruction of the object. However, this method still has some drawbacks. The time taken by the reconstruction algorithm can become very long for large images and one needs to compute the speckle pattern at the object position by using the Fresnel-Huygens propagator on the phase pattern imprinted on the SLM. While the latter is not a big problem - one can calculate the speckle pattern once and reuse the phase pattern masks - the computational overhead, given by the computational effort once all measurements have been made, is a huge problem. The overhead becomes especially problematic considering typical problems in spectroscopy (e.g. pump-probe spectroscopy), where many similar images need to be taken, while one experimental parameter is changed. Here, it is desirable to have the reconstructed image directly, so one can use this information when taking the next image. For example one could adaptively scan the previous image for regions of large values or strongly varying values and scan these areas with higher resolution in the next image.

## Results

### The adaptive compressive CGI algorithm

**Figure 1:**
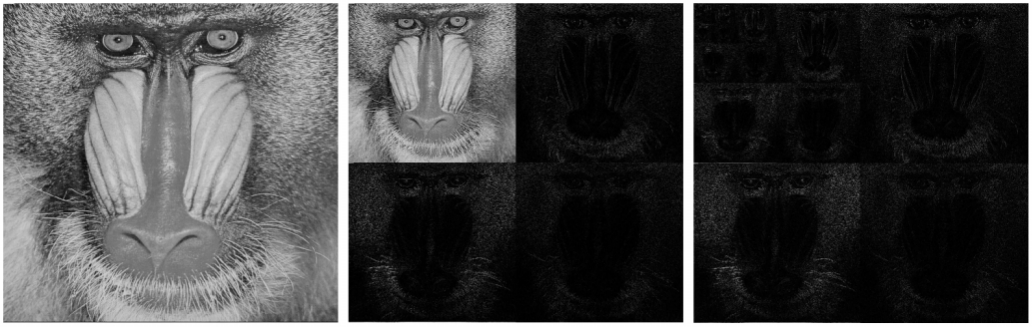
512 × 512 pixel baboon test image (left panel) and its one-step (middle panel) and complete (right panel) wavelet transform. For the transform absolute values of the wavelet coefficients are shown. White regions correspond to large wavelet values and mark regions with strong edges. Every wavelet coefficient at scale *j* contains information about four pixels of the coarse image of size 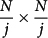. Also, every wavelet coefficient has four children wavelet coefficients at scale *j* − 1 which contain information about the same range of the image.

We demonstrate an alternative way to perform compressive CGI (CCGI) without any computational overhead once all measurements have been performed by using an adaptive measurement scheme. We follow a promising strategy for adaptive compressive sensing that suggests replacing the random speckle patterns by directly using the patterns that form the sparse basis^13^. We start the discussion of our strategy by recalling the properties of the 2D Haar wavelet transform of square images consisting of *N* × *N* pixels. The wavelet decomposition procedure is schematically depicted in figure 1. The decomposition of the image *I* (*x, y*) is performed seperately for rows and columns. At first each row is divided into 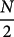 pairs of adjacent pixels. The partial wavelet transform *T*′(*x, y*) now consists of the sum and the difference of these adjacent pixels according to the following rules for 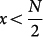: 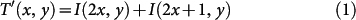

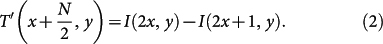


Repeating that procedure for each column in *T*′ according to similar rules for 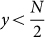 gives the full transform *T*(*x, y*): 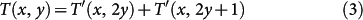

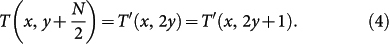


The resulting transform now consists of four quadrants. The upper left quadrant represents a coarse version of the original image, while the other three quadrants contain information about horizontal, vertical and diagonal edges. One may now continue and perform another wavelet transform on the upper left quadrant and iteratively repeat this procedure until the pixel in the upper left corner contains the mean intensity of the picture and all other pixels contain information about edges. Now each additional transform performed corresponds to a coarser scale *j* with wavelet coefficients spanning over larger regions, but carrying information over a smaller range of frequencies. Such wavelet representations are efficient in terms of image compression. Only a small portion of natural images consists of edges and only wavelet coefficients corresponding to regions with sharp edges are large, therefore only few large coefficients are sufficient to approximate the full image. As can be seen in figure 1, the number of large wavelet coefficients (shown in white) is rather small.

**Figure 2:**
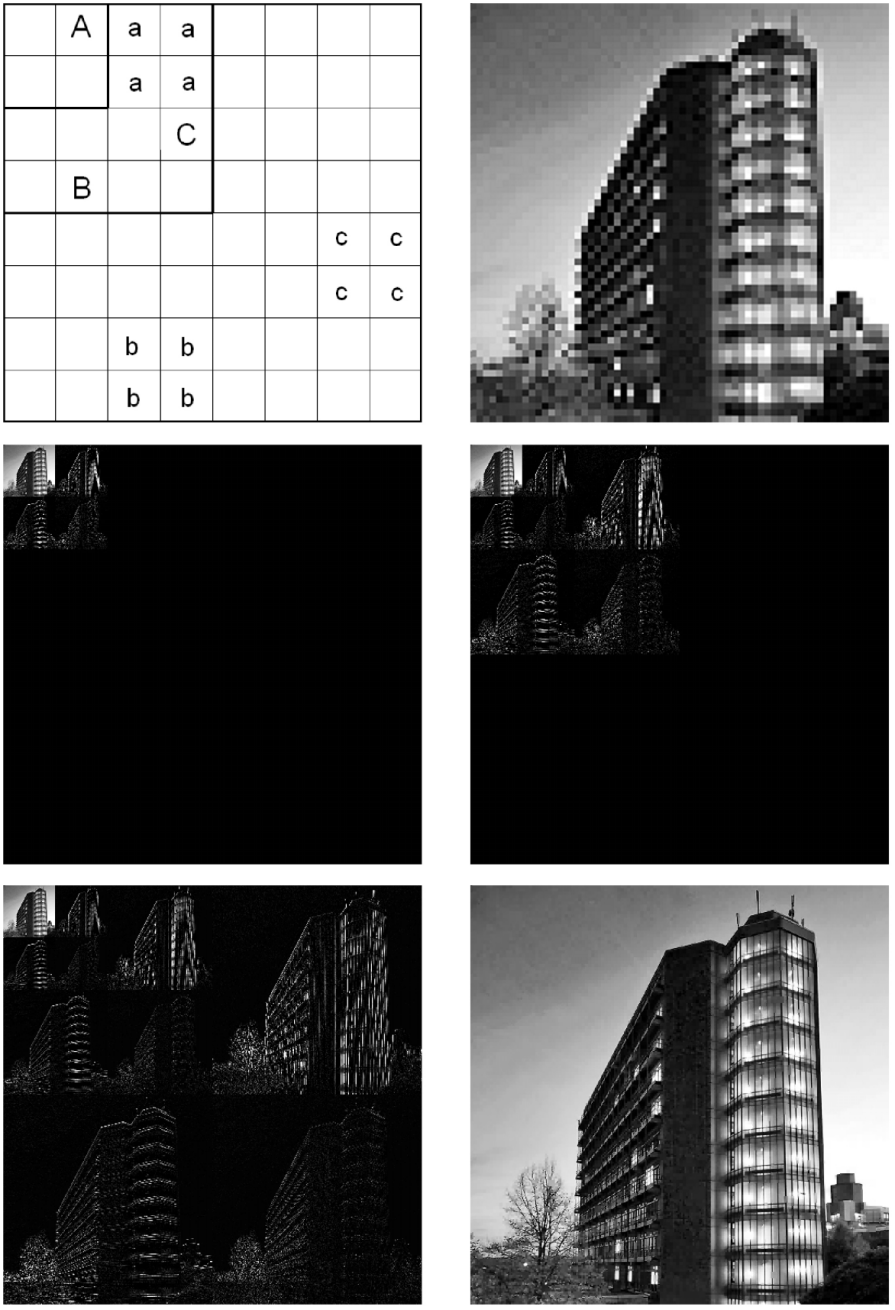
Summary of the CCGI scheme. First, a low resolution real space image is taken (upper right panel).The wavelet transform of that image is created (middle left panel). Large wavelet coefficients are shown in white, small ones in black. For each wavelet coefficient larger than the chosen threshold, its four children coefficients are determined. See the upper left panel for exemplaric parent (capital letters) and corresponding children wavelets (corresponding lower case letters) across different scales. The measurement of a children wavelet coefficient requires four real space measurements at a finer scale. After all target wavelet coefficients at the finer scale are measured (middle right panel), the procedure continues with the next finer scale until the target scale *j* = 1 is reached or no wavelet coefficient is larger than the threshold value (lower left panel). The result is then converted back to a real space image using the inverse wavelet transform (lower right image). For this example the number of measurements needed is roughly 40% of the number of pixels present in the image. Note that the upper right, lower left and lower right sector of the wavelet transform correspond to horizontal, vertical and diagonal edges, respectively. Wavelet coefficients have been multiplied by 8 to enhance contrast.

This strategy becomes interesting as the wavelet transformation is hierarchic. Every parent coefficient at some coarse scale has four children coefficients at the next finer scale covering the same spatial region. As it is very likely that the children wavelet coefficients belonging to parent coefficients which are small will also be small, this offers a great opportunity for image compression in terms of wavelet trees^14^ by cutting of these trees at an adequate scale. We follow a similar strategy and first take a coarse image of size 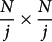. Experimentally, this is realized by inserting a phase-only SLM (Holoeye-Pluto) in the path of a laser beam polarized such that the SLM only introduces a phase shift to it. The phase pattern imprinted on the SLM is the Fourier transform of a square superposed with the phase map of a lens. As a consequence, in the focal plane behind the SLM the square is recovered in the spatial intensity pattern of the light beam. We precomputed 87040 of such phase patterns using an iterative numerical technique based on the adaptive-additive algorithm^15^. 65536 of these form the pixels of a 256 × 256 (*j* = 1) pixel square. The other patterns form the pixels of squares of the same size, but consisting of fewer (128 × 128 (*j* = 2), 64 × 64 (*j* = 3) and 32 × 32 (*j* = 4)), but larger pixels of size 2^(2(*j*−1))^, respectively. The object to be imaged is placed at the focal plane of the SLM (*f* = 36 cm) and the transmission through that object is measured. Under the conditions used throughout the manuscript, the whole square has a side length of 32 mm. When the coarse image is taken, we perform a one-step wavelet transform on it. Now we check the absolute values of the wavelet coefficients corresponding to edges against a predefined threshold *I_j_*. If the values are larger than *I_j_*, the four children wavelet values at the next finer scale *j* − 1 are measured too. As each wavelet coefficient spans over exactly four pixels at its scale, it is never necessary to perform more than four measurements in real space to determine any wavelet value. Once all the measurements at the finer scale have been performed, a new finer image can be constructed. It consists of the newly measured transmission values for regions containing sharp edges and of the transmission values already measured at a coarser scale for regions without edges. Now another one-step wavelet transform is performed on this finer image and again all wavelet values are checked against a new threshold *I_j_*_−1_. This process is repeated until the finest picture at scale *j* = 1 is constructed. A summary of the imaging process is presented in fig. 2.

### Experimental results

**Figure 3:**
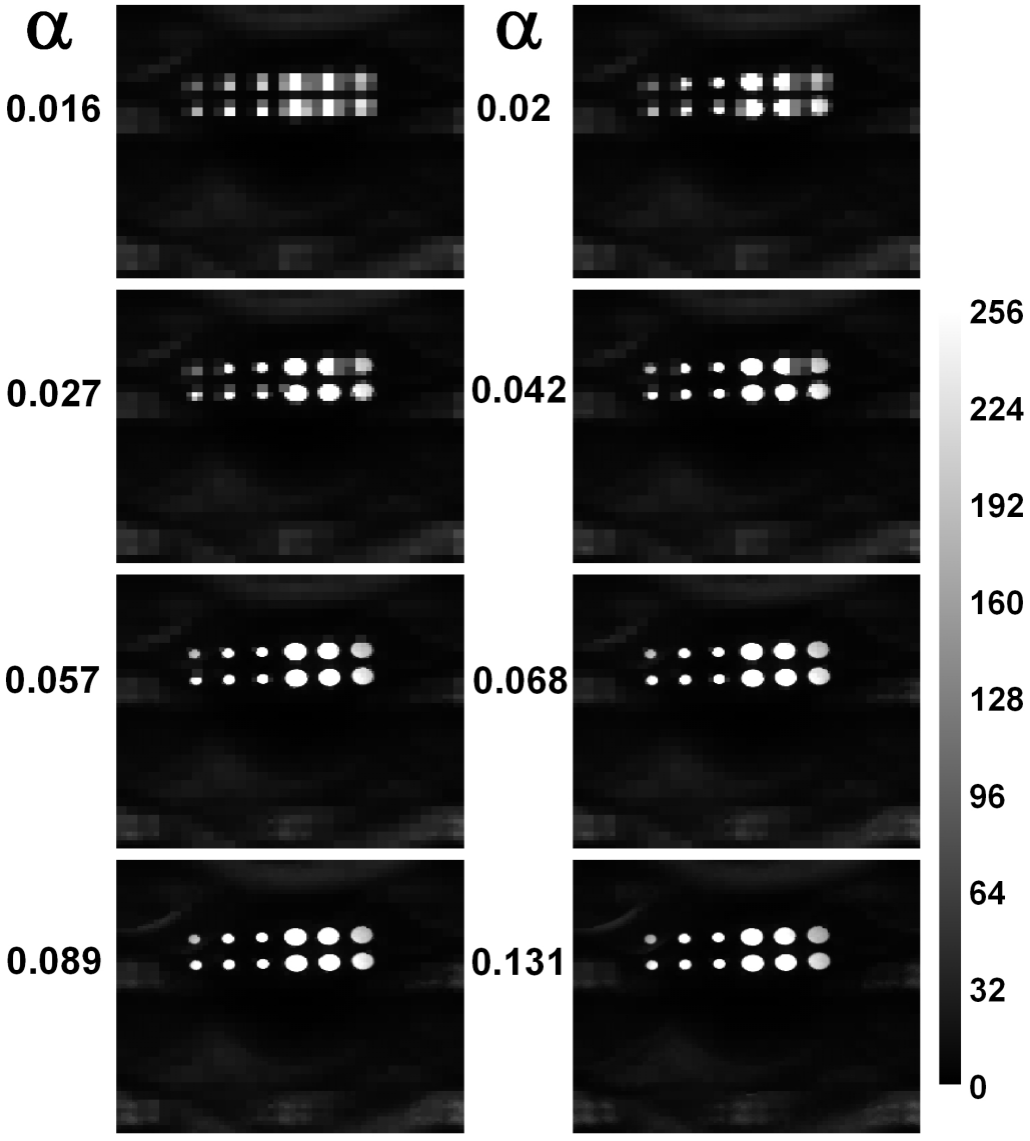
Normalized transmission maps through a metal plate containing twelve holes. The large holes have a diameter of 2 mm, while the smaller ones have a diameter of 1 mm. *α* gives the decreased acquisition rate. A faithful image of the plate is already possible with approximately 5–7% of the measurements required to record every single pixel in full resolution.

**Figure 4:**
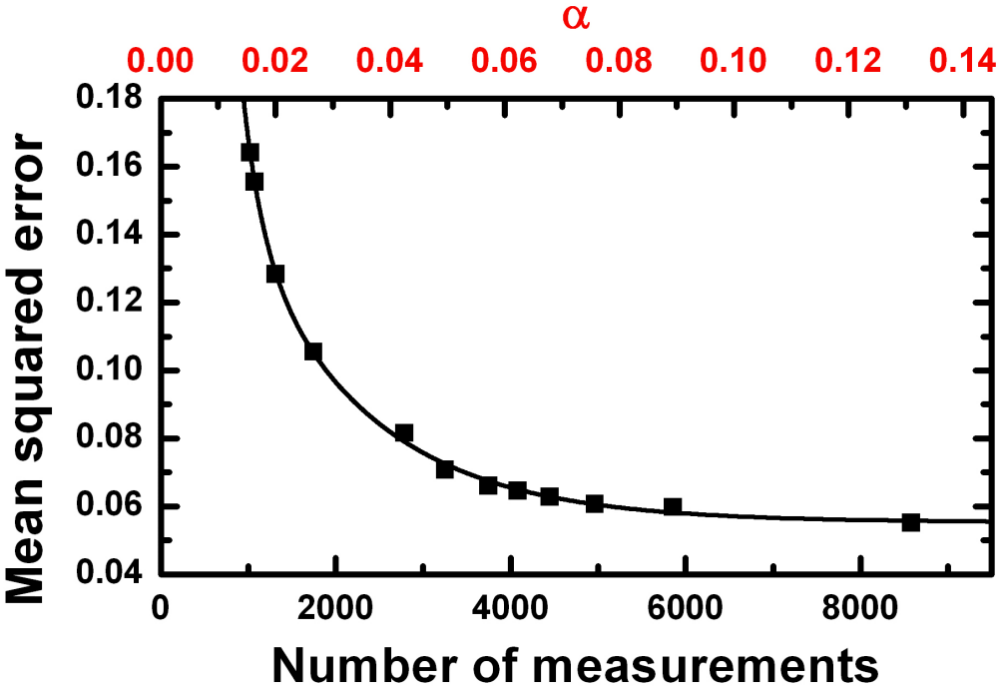
Mean squared error versus the number of measurements taken. The residual error saturates for *α* > 7%.

**Figure 5:**
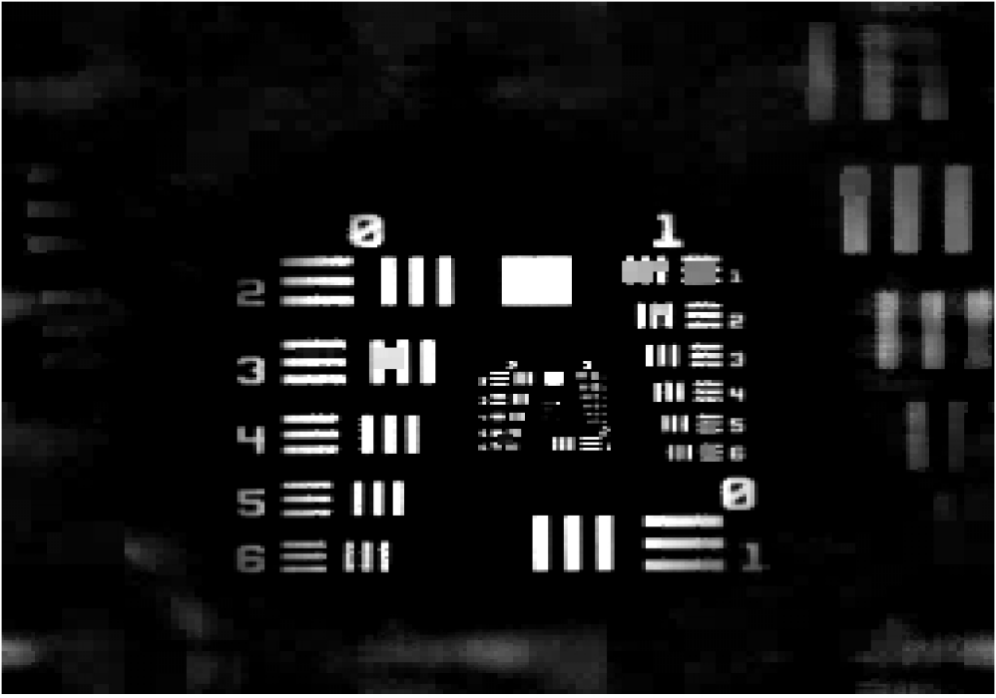
Normalized transmission map through a 1951 USAF resolution test chart at *α* = 0.369.

We tested the CCGI algorithm using a metal plate containing twelve holes as a test target. We chose to use a threshold which becomes sharper at finer scales (*I_j_*_−1_ = 2*I_j_*) and varied the initial threshold *I*_4_, resulting in images of differing quality. The results are shown in figure 3. Here the transmission maps quantized to 256 greyscales are shown in terms of the decreased acquisition rate *α*, which is the total number of measurements performed on all scales divided by the total number of pixels present on the finest scale (*N*^2^ = 65536). The transmission is normalized to the empty space transmission to account for possible inhomogeneities introduced by the SLM. As can be seen, the image is reproduced quite well at relatively small *α*. At around 2% the quality is already sufficient for distinguishing the holes and counting their number. For *α* around 4% the image already looks reasonable. For *α* around 7% the recorded image shows good quality. For larger *α* only small improvements are seen. However, to get a more quantitative measure of the recorded image quality, we calculated the mean squared error 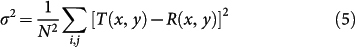
of the measured image as compared to the reference construction drawing *R*(*x, y*) of the metal plate containing the holes. Results are shown in figure 4. The impression that the image quality does not improve significantly for *α* > 7% is verified. The mean squared error roughly follows an exponential decay and saturates approximately at a value of 0.055 for large *α*. A closer examination of this residual error shows that it is mainly caused by the edges of the holes. In contrast to the construction drawing, the edges between full transmission and zero transmission are not positioned at pixel borders. Therefore the pixels at the edges show some intermediate transmission and introduce some deviations from the reference image. The number of necessary measurements needed for near optimal reconstruction of an image obviously depends on the number of large wavelet coefficients that image carries. In order to demonstrate the adaptivity of our technique, we kept the threshold setting used for measuring the metal plate at *α* = 0.131 in figure 3 and imaged a more complex object - a 1951 USAF resolution test chart. The recorded image is shown in figure 5. The image quality is still good, but the algorithm automatically took almost three times more measurements than were needed for the metal plate, resulting in *α* = 36.9%. The image resolution is reasonable. One pixel has a length of about 125 *μ*m in real space which is roughly the size of the lines on the test plate which can still be resolved. Nevertheless the image shows some artifacts. These are a consequence of a weakness of the algorithm used. Strictly periodic structures like several parallel lines placed next to each other may look like having no edge at all at a coarser scale. However, such problems may be overcome by more advanced algorithms relying on taking more than just the parent wavelet value into account when deciding on whether a certain wavelet value should be measured or not^13^.

Our technique offers a wide range of advantages. As it is adaptive, one has control over the quality of the image in advance by choosing the thresholds. The algorithm does not require any additional computationally intensive recovery algorithms needed for standard compressive sensing techniques using pseudorandom illumination patterns. Our technique works reasonable fast. The SLM can be operated at up to 60 Hz. Photodiodes and readout circuitry working on the same timescale are common today, opening up the possibility to record images of reasonable resolution and quality within few minutes. In particular experimental techniques requiring single pixel detectors like lock-in detection for sensitive pump-probe measurements may benefit from our results. Spatially resolved measurements are to the best of our knowledge not carried out with high resolution using such techniques due to the long measurement durations that would be needed. Reducing the number of necessary measurements by a factor of at least fifteen opens up the way to perform such measurements with high spatial resolution. If the duration of the measurement is more crucial than the image quality, our approach also allows one to perform a fixed number of finer measurements for a preset number of largest wavelet coefficients at each scale instead of using thresholds. In that way it is possible to take an image in a fixed amount of time.

## Discussion

In conclusion we have developed an adaptive CCGI technique that allows us to record images using a single pixel camera at an acquisition rate fifteen to twenty times below the Shannon limit by recording the image directly in a sparse basis. A number of further research directions arise from our work. Compressive imaging techniques are not limited to recording image information, but have also found usage far beyond simple imaging applications in fields like quantum process tomography^16^,^17^, optical encryption^18^, fluorescence microscopy and hyperspectral imaging^19^. Also our approach is quite generic. Optimized approaches which also take the magnitude of neighboring wavelet coefficients into account^13^ instead of just the parent wavelet coefficient may lead to increased image quality or smaller values of *α*. Also, it is well known that especially designed measurement matrices can drastically reduce the number of needed measurements for exact image reconstruction using seeded belief propagation techniques^20^. Finally, it is not strictly necessary to use precomputed phase patterns, but one could compute them on the fly, thereby allowing one to even choose an adaptive wavelet basis. Yet, the greatest strength of our approach lies in drastically reducing the needed measurement time for high-resolution images using single-pixel detectors without having any need for computational image reconstruction.

We would like to conclude this paper by a comparison between CCGI and standard random Gaussian matrix based compressive sensing techniques (RMCS) to identify the strengths and weaknesses of our approach in more detail. Obviously, having the image available once all the measurements are taken, is an advantage, but it is also introduces a drawback: CCGI needs to use a predefined sparse basis, while RMCS will automatically find an adequate sparse basis during reconstruction. Accordingly, it is typically possible to achieve a near-ideal reconstruction with fewer measurements using RMCS. However, it should be noted that the exact number of measurements needed for near-ideal reconstruction is usually not known a priori as it depends on the sparsity of the image. The needed number of measurements is also unknown in CCGI, but specifying the desired image quality in terms of the threshold *I_j_* ensures that not too many measurements are made. The exact number of measurements needed for CCGI is hard to predict as it depends on how sparse an image is in the wavelength basis. For very sparse images, the penalty can be as much as 50%. For less sparse images, the differences are less drastic. However, for a comparable number of measurements, CCGI-based methods tend to achieve a better signal to noise ration than RMCS methods do. See Ref. 13 for a detailed comparison of a slightly modified version of CCGI with state of the art RMCS techniques. Another important benchmark is the performance of compressive sensing techniques in the presence of noise. In CCGI noise can become a severe problem if the noise magnitude becomes comparable to the threshold chosen. CCGI is therefore not the method of choice for measuring images containing strong noise or weak signals. Another issue is scalabilty. Going to larger images, increases the necessary number of measurements a computations during data acquisition in CCGI and the complexity of the minimization problem in RMCS. However, for all the image sizes we examined, the time needed for performing the measurements was always so much longer than the time needed for performing the wavelet transforms and building the sampling queue that no delay was noticeable. In summary, although other compressive sensing methods based on Gaussian random matrices approaches typically need fewer measurements than most (but not all^21^) techniques using deterministic matrices, having the result immediately renders adaptive spatially resolved pump-probe spectroscopy and other delicate spectroscopic techniques with high resolution possible. Therefore, we suggest that our technique is well suited for specialized complex problems in physics and spectroscopy which are a priori known to be reasonably sparse in the wavelet basis, while RMCS methods are a much better choice for taking single images, for images where noise is an issue and for images where the sparsest basis is unknown.

## Methods

The objects to be imaged were placed at the focal plane of the SLM (*f* = 36 cm) and the transmission through the objects was measured by a standard commercial photo diode onto which the transmission through the object was focused and a Keithley 2000 multimeter was used for measuring the photo diode output. The hole test plate used consisted of twelve holes. Six of these holes had a diameter of 2 mm, while the other six holes had a diameter of 1 mm. Their average separation was around 1.5 mm. The laser used for the transmission measurements was a pulsed Ti:Sapph laser emitting pulses wih a duration of approximately 2 ps at a wavelength of 800 nm.

## Author Contributions

M.A. designed the experiment and analyzed the data. M.A. and M.B. wrote the manuscript.
